# COVID-19 Evidence Accelerator: A parallel analysis to describe the use of Hydroxychloroquine with or without Azithromycin among hospitalized COVID-19 patients

**DOI:** 10.1371/journal.pone.0248128

**Published:** 2021-03-17

**Authors:** Mark Stewart, Carla Rodriguez-Watson, Adem Albayrak, Julius Asubonteng, Andrew Belli, Thomas Brown, Kelly Cho, Ritankar Das, Elizabeth Eldridge, Nicolle Gatto, Alice Gelman, Hanna Gerlovin, Stuart L. Goldberg, Eric Hansen, Jonathan Hirsch, Yuk-Lam Ho, Andrew Ip, Monika Izano, Jason Jones, Amy C. Justice, Reyna Klesh, Seth Kuranz, Carson Lam, Qingqing Mao, Samson Mataraso, Robertino Mera, Daniel C. Posner, Jeremy A. Rassen, Anna Siefkas, Andrew Schrag, Georgia Tourassi, Andrew Weckstein, Frank Wolf, Amar Bhat, Susan Winckler, Ellen V. Sigal, Jeff Allen

**Affiliations:** 1 Friends of Cancer Research, Washington, District of Columbia, United States of America; 2 Reagan-Udall Foundation for the FDA, Washington, District of Columbia, United States of America; 3 Health Catalyst, Salt Lake City, Utah, United States of America; 4 Gilead Science, Inc. Foster City, California, United States of America; 5 COTA, Inc., Boston, Massachusetts, United States of America; 6 Syapse, San Francisco, California, United States of America; 7 Massachusetts Veterans Epidemiology Research and Information Center (MAVERIC), VA Boston Healthcare System, Boston, Massachusetts, United States of America; 8 Department of Medicine, Brigham and Women’s Hospital, Harvard Medical School, Boston, Massachusetts, United States of America; 9 Dascena, Oakland, California, United States of America; 10 Aetion, New York, New York, United States of America; 11 Division of Outcomes and Value Research, John Theurer Cancer Center at Hackensack University Medical Center, Hackensack, New Jersey, United States of America; 12 VA Connecticut Healthcare System, West Haven, Connecticut, United States of America; 13 Yale University Schools of Medicine and Public Health, New Haven, Connecticut, United States of America; 14 HealthVerity, Philadelphia, Pennsylvania, United States of America; 15 TriNetX, Cambridge, Massachusetts, United States of America; 16 National Center for Computational Sciences Oak Ridge National Laboratory, Oak Ridge, Tennessee, United States of America; National Institute for Infectious Diseases Lazzaro Spallanzani-IRCCS, ITALY

## Abstract

**Background:**

The COVID-19 pandemic remains a significant global threat. However, despite urgent need, there remains uncertainty surrounding best practices for pharmaceutical interventions to treat COVID-19. In particular, conflicting evidence has emerged surrounding the use of hydroxychloroquine and azithromycin, alone or in combination, for COVID-19. The COVID-19 Evidence Accelerator convened by the Reagan-Udall Foundation for the FDA, in collaboration with Friends of Cancer Research, assembled experts from the health systems research, regulatory science, data science, and epidemiology to participate in a large parallel analysis of different data sets to further explore the effectiveness of these treatments.

**Methods:**

Electronic health record (EHR) and claims data were extracted from seven separate databases. Parallel analyses were undertaken on data extracted from each source. Each analysis examined time to mortality in hospitalized patients treated with hydroxychloroquine, azithromycin, and the two in combination as compared to patients not treated with either drug. Cox proportional hazards models were used, and propensity score methods were undertaken to adjust for confounding. Frequencies of adverse events in each treatment group were also examined.

**Results:**

Neither hydroxychloroquine nor azithromycin, alone or in combination, were significantly associated with time to mortality among hospitalized COVID-19 patients. No treatment groups appeared to have an elevated risk of adverse events.

**Conclusion:**

Administration of hydroxychloroquine, azithromycin, and their combination appeared to have no effect on time to mortality in hospitalized COVID-19 patients. Continued research is needed to clarify best practices surrounding treatment of COVID-19.

## Background

Despite a growing body of literature about COVID-19 and its cause, SARS-CoV-2, much remains unclear about which treatment strategies are most effective for the entire clinical course of the disease. Several therapeutic agents have been investigated, including the anti-malarial drug hydroxychloroquine. The evidence regarding its use for the treatment of COVID-19 continues to evolve [1, 2].

The use of hydroxychloroquine for COVID-19 was initially supported by *in vitro* studies showing anti-inflammatory and anti-SARS-CoV-2 activity [3]. Methodological weaknesses have marked subsequent *in vivo* studies; many studies have enrolled small numbers of patients and have not included appropriate control groups or methods to control for confounding variables, making interpretation of findings difficult [4]. While some non-randomized studies have shown a survival benefit for COVID-19 patients receiving hydroxychloroquine [5], others have found no evidence of benefit [6]. Still others have identified safety concerns, including an increased risk of prolonged QT intervals and arrhythmias for patients receiving hydroxychloroquine [7–9]. Additional studies have found that hydroxychloroquine may be more effective when given in combination with azithromycin [5] while emerging experience may indicate otherwise, leading to further uncertainty about the appropriate use of hydroxychloroquine. Differing methodologies to control for potential bias, incomplete capture of the timing of mechanical ventilation in relation to receipt of hydroxychloroquine, may have contributed to these inconsistencies.

Despite the development of treatment guidelines for COVID-19 [10], significant questions remain about best treatment practices. COVID-19 remains a significant global threat, and epidemiologic models have predicted that transmission will continue through the coming years [11]. Due to the significant morbidity and mortality associated with severe cases of COVID-19, establishing treatment guidelines is an essential step towards improving patient outcomes. It is therefore important to address methodological inconsistencies of existing studies and remaining uncertainties about the efficacy of hydroxychloroquine for the treatment of COVID-19.

Towards this end, the COVID-19 Evidence Accelerator convened by the Reagan-Udall Foundation for the FDA, in collaboration with Friends of Cancer Research, assembles experts from the health systems research, regulatory science, data science, and epidemiology to participate in parallel analyses. Analytic partners align on a common protocol and conduct analyses independently; methods and results are shared side-by-side to evaluate differences and similarities. Results are presented to a larger audience, including experts and leaders from the FDA, to provide informal discussion and review. Several groups, representing distinct populations within the U.S. to conduct parallel analyses of the effect of hydroxychloroquine, azithromycin, and the two drugs in combination on COVID-19 outcomes to compare results and better understand differences in the safety of these treatments for COVID-19.

## Methods

### Ethical statement

All the data partners received Institutional Review Board (IRB) approval or exemption. The use of VA data was approved by both the Department of Energy (DOE) (Oak Ridge Sitewide IRB00000547 for Protocol ORAU000718) and VA review committees and engages both VA and DOE researchers (VA-DOE Reliance Agreement under the authority of 38 U.S.C. 7303 and 38 U.S.C. 523). In addition to IRB approval, VA R&DC reviewed research proposals for final Institutional approval and ensured that all research in which the facility is engaged is consistent with the VA mission and complies with all applicable statutory and regulatory requirements. A Waiver of HIPAA Authorization and a Waiver of Informed Consent were approved for this study ORAU000718. The Aetion/HealthVerity study was approved under exemption by the New England Institutional Review Board (protocol #1-9757-1) and received a waiver of informed consent. The COTA study received approval by the Hackensack Meridian Health IRB (Pro2020-0342) and received a waiver of informed consent. The Health Catalyst dataset used for this analysis has been de-identified following the expert determination method outlined in 45 CFR 164.514(b)(1). Health Catalyst uses an external vendor to certify that the dataset is de-identified in accordance with 45 CFR 164.514(b)(1). The Dascena study received approval from the Pearl Institutional Review Board (20-DASC-120) and was granted a waiver of informed consent. The TriNetX Platform receives Protected Healthcare Information (PHI) or a Limited Data Set (LDS) from Healthcare Organizations (HCO) strictly under the constraints defined in a Business Associate Agreement (BAA) or a Data Use Agreement (DUA) under the United States (U.S.) Health Insurance Portability and Accountability Act (HIPAA). A fundamental Data Privacy principle is that TriNetX does not expose PHI or LDS to the end users of the TriNetX Platform. The data made available from the TriNetX platform is de-identified based on standard defined in Section §164.514(a) of the HIPAA Privacy Rule. The process by which Data Sets are de-identified is attested to through a formal determination by a qualified expert as defined in Section §164.514(b)(1) of the HIPAA Privacy Rule. Sypase conducted this work through a Research Collaboration Agreement with the FDA to include an IRB exemption through the Office of the Chief Scientist (OCS) Human Subject Protection (HSP) Executive Officer and all of this work involved data from secondary sources. The RCA work has been performed under an exemption from the Office of the Chief Scientist (OCS) Human Subject Protection (HSP) Executive Officer at FDA.

### Data sources

**Fig 1:**
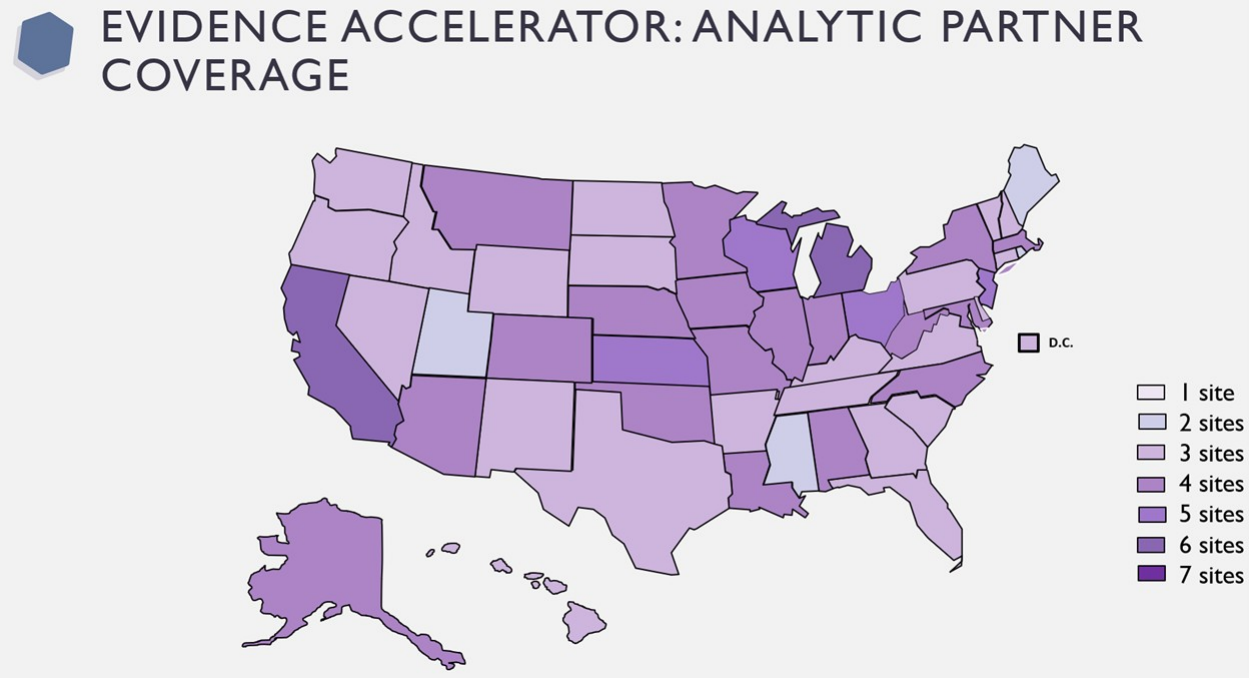
Partner map for HCQ analysis. The Evidence Accelerator partnered with seven groups to conduct the parallel analyses. This is a graphical description of coverage and overlap for each group conducting the parallel analysis and their distinct sources. Republished from https://www.brightcarbon.com/resources/editable-powerpoint-maps/ under a CC BY license, with permission from Bright Carbon, original copyright 2020.

The Evidence Accelerator partnered with seven groups to conduct the parallel analyses: Syapse, COTA/Hackensack Meridian Health (HMH), Dascena, TriNetX, Health Catalyst, Aetion, and Veteran’s Health Administration (VA). Each group conducting the parallel analysis collected data from their distinct sources. Syapse, COTA, Dascena, TriNetX, Health Catalyst and VA all utilized electronic health record (EHR) data, while Aetion utilized medical and pharmacy claims, and hospital billing data drawn from the HealthVerity Marketplace. Syapse utilized the EHR and molecular diagnostic lab information from two large Midwestern US health systems. COTA utilized data from the Real-world Evidence COVid RegistrY (RE-COV-RY) database collected at Hackensack Meridian Health System. Dascena utilized data from the EHRs from eight US hospitals. TriNetX drew data from the TriNetX Dataworks USA Network. Health Catalyst drew data from 17 Health Catalyst clients; the group had access to EHR data including medication administration. Aetion drew data from the HealthVerity linked medical and pharmacy claims, labs, and hospital chargemaster dataset. The VA used EHR data from the national VA Healthcare System, with COVID-19 cases adjudicated through a National Surveillance Tool [12]. A graphical description of coverage and overlap is illustrated in Fig 1 and characteristics of participating data sources and populations is described in Table 1.

**Table 1:** Characteristics of participating data sources and populations.

Study Variable	Aetion	COTA	Dascena	Health Catalyst	Syapse	TriNetX	VA Dataset
**Data source**	HealthVerity Medical and Pharmacy Claims and Hospital Chargemaster	Hackensack Meridian Health System	Electronic health records from eight US hospitals	17 Health Catalyst clients	Integrated data from health systems (e.g. electronic medical records, ancillary clinical sources) and molecular diagnostic labs	TriNetX Dataworks USA Network	Veteran’s affairs healthcare electronic health records. Covers all VA-hospitals with over 9 million active users.
				No claims, pharmacy data available			
		HMH Real-world Evidence COVid-RegistrY (RE-COV-RY)					
				EMR data: medication administrations, prescriptions, labs, diagnoses			
**Region**	Coverage across the U.S. Majority (57%) in the Northeast Region.	Northeast Region; New Jersey		Across the U.S. with coverage in 24 states	Midwest	Across the Northeastern, Southern, Midwestern, and Western regions of the US.	Nationwide sample representative of COVID pandemic geographic distribution as of study time period.
**Data processing**	Open claims data from data clearing houses. Chargemaster data from hospital billing system (with details of hospital stays). Lab data from a major lab provider (Quest) plus several smaller providers. The data is brought together by the HealthVerity de-id engine through a common patient ID.	EHR data abstracted through chart review into REDCap		Data from multiple EMRs standardized into single data model; additional regular expression and curation performed to identify COVID labs	Data from clinical systems at multiple health systems and molecular data from diagnostic labs integrated into a common data model	Various and disparate data is mapped to industry standard terminologies which produces master terminology/intelligent synonym search.	Corporate data warehouse (CDW) repository of EHR for entire VA. Consists of inpatient and outpatient diagnosis codes, laboratory results, pharmacy prescription fills (inpatient, outpatient, and IV), vitals, health factors, and demographic data.
**Data collection period**	February 2020-April 2020 with follow up through May 2020	March 2020—May 2020	March 1, 2020-June 20, 2020	Mar 2020—May 2020	February 1 –June 13, 2020	All patients were diagnosed with COVID-19 and hospitalized between January 20, 2020 and April 27th 2020.	Case-positive individuals as of April 30, 2020 followed through EHR data as of July 14, 2020.

### Patient inclusions

**Fig 2:**
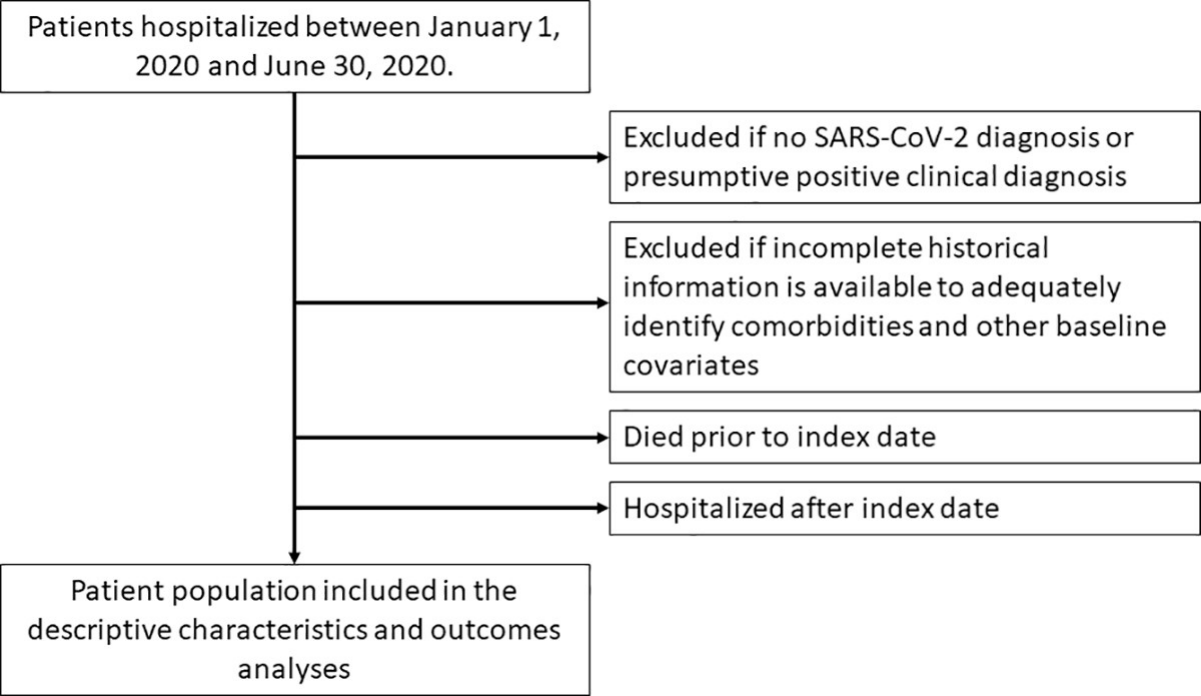
Study diagram. Common exclusion criteria were applied across the datasets to arrive at a final study population to assess patient characteristics and treatment outcomes for patients with COVID-19.

Data were gathered from the 7 EHR datasets for hospital admissions in the U.S. between January 1, 2020 and June 30, 2020 (index hospitalization). Patients were eligible for inclusion if they had tested positive for COVID-19 during or prior to their visit or had an International Classification of Diseases (ICD)-10 code for COVID-19 in the 21 days leading up to admission, during admission or as a discharge diagnosis (Fig 2). All groups using ICD codes considered ICD-10 code U07.1; Syapse, Dascena and COTA additionally considered codes B97.21, B97.29, J12.81, B34.2; for the primary results Health Catalyst required a discharge diagnosis of U07.1 either primary or secondary to a related primary diagnoses (e.g., pneumonia or acute respiratory distress syndrome). To ensure accurate assessment of comorbidities, Syapse imposed a one-year minimum enrollment criteria to their study population of patients diagnosed with malignant cancers on or after January 1, 2015.

All patient data was maintained in compliance with the Health Insurance Portability and Accountability Act (HIPAA).

### Treatment

Patients were considered to be treated with hydroxychloroquine and/or azithromycin if they received any of those medications at any point during their hospitalization, before discharge or death (the VA only considered treatments that occurred in the first 48 hours following hospitalization; Health Catalyst required treatment initiation within the first 2 days following hospitalization). Three treatment groups were compared to the population of patients that received neither hydroxychloroquine nor azithromycin. These treatment groups were hydroxychloroquine and azithromycin in combination, hydroxychloroquine alone, and azithromycin alone. Groups varied in their approach to determining date of cohort entry and index date of treatment (see S1 File for details). This study was non-interventional and treatment groups were not randomized.

### Covariates

For each patient, data on potential demographic, medication and health-related covariates were extracted from structured EHR fields, prescription dates, and ICD-10 codes. In one analysis data were also extracted from structured and unstructured hospital billing data. In two networks, this data was augmented with claims data. A subset of participating groups adjusted for health-related variables and medication use prior to the index hospitalization, sociodemographic factors and comorbid conditions. The ICD codes used to identify comorbidities are presented in the S1 File. Covariates were selected independently by each group. Covariates considered by each group, and the method of their selection, are presented in Table 2.

**Table 2:** Covariates reported by each group.

	Health-related Variables	Medication Usage	Sociodemographic variables	Confounder selection method
**Aetion**	• Baseline health status: chronic comorbidities, lifestyle factors, health resource utilization • Pre-admission confounders related to COVID-19 severity: pre-admission symptoms, pre-admission health resource utilization, no. days since symptom onset • Admitting characteristics: Hospital characteristics (e.g. urban vs rural, no. of beds, teaching status), admitting status, admitting diagnoses • In-hospital confounders: COVID-19 severity at treatment, trajectory in severity, respiratory support and procedural treatments, ICU utilization	• Baseline medication use: chronic medication use, no. of unique medications dispensed, no. prescriptions dispensed • COVID-19-related medications: pre-admission outpatient treatments, inpatient antithrombotics, inpatient antivirals/antibiotics, other experimental COVID-19 therapies administered prior to or concurrent with treatment index date, time from admission to treatment	• Age • Sex • Calendar month of cohort entry • Insurance type • US region	A priori assumptions about confounding structure. and prior literature
**COTA Hackensack Meridian Health**	• Smoking history • Number of comorbidities at baseline • Comorbid diagnoses • ICU status • Fever • Respiratory rate • C-reactive protein • Oxygenation status • qSOFA	Insulin	• Age • Sex • Race • Nursing home status prior to admission	Lasso regression using 5-fold cross validation, with priority given to variables significant in determining the outcome of interest
**Dascena**	• Vital signs and lab values at admission (oxygen saturation, D-Dimer, lactate, temperature, white blood cell count, respiratory rate, heart rate, and systolic blood pressure) • Comorbid diagnoses	remdesivir, macrolide antibiotics, angiotensin receptor blockers (ARB), angiotensin-converting enzyme (ACE) inhibitors, non-steroidal anti-inflammatory drugs (NSAID), steroids, tocilizumab, and statins	• Age • Sex • Race/ethnicity • Income	A priori assumptions about confounding structure and prior literature
**Health Catalyst**	• Chronic comorbid diagnoses • History of supplemental oxygen use • History with health-related behaviors (e.g., smoking)	• remdesivir, macrolide antibiotics, angiotensin receptor blockers (ARB), angiotensin-converting enzyme (ACE) inhibitors, non-steroidal anti-inflammatory drugs (NSAID), steroids, tocilizumab, and statins	• Age • Sex • Race/ethnicity • Income	A priori assumptions about confounding structure and prior literature coupled with empiric interrogation.
**Syapse**	• Chronic Comorbidities Clinical characteristics at hospital admission	Not Reported	• Age • Sex • Race/ethnicity • Estimated Income	A priori assumptions about confounding structure and prior literature.
**TriNetX**	• Comorbid diagnoses • Oxygenation status • Baseline invasive	• angiotensin-converting enzyme (ACE) • Angiotensin receptor blockers	• Age • Sex • Race • Ethnicity	A priori assumptions about confounding structure and prior literature
**VA**	• Lab orders (Lactate dehydrogenase, C-reactive protein. D-dimer, Ferritin) • Height and weight • Smoking status and alcohol use • Concurrent inpatient treatments • Chronic comorbidities • Frailty • Lab results and vital signs	• Chronic medication use • Concurrent inpatient treatments	• Age • Sex • Urbanicity • Region of US • Long-term care status • Calendar week of admission • Station size or number of veterans in care	A priori assumptions about confounding structure and prior literature.

### Outcomes

Three primary outcomes were measured: use of mechanical ventilations (as a potential indicator of overall health status of patients), evidence of benefit of hydroxychloroquine (determined by hospital discharge as an indicator of recovery), and in-hospital mortality (determined by discharge disposition). Measurement of overall health status and evidence of benefit varies across groups, contingent on data availability. A summary of outcome definitions is presented in Table 3.

**Table 3:** Summary of outcome definitions across groups.

Group	Mechanical Ventilation	Evidence of Benefit
**Aetion**	Mechanical ventilation	Not assessed
**COTA Hackensack Meridian Health**	Mechanical Ventilation	Hospital Discharge
**Dascena**	Mechanical Ventilation	Hospital Discharge
**Health Catalyst**	Not assessed	Not assessed
**Syapse**	Mechanical Ventilation	Not assessed
**TriNetX**	Not assessed	Improvement from “hospitalized with any oxygen support” to either “hospitalized on room air” or “discharge” following the index date
**VA**	Mechanical Ventilation within 21 days	Not assessed

We also assessed the proportion of patients in each treatment group experiencing any of the following adverse events: diarrhea, hypoglycemia, cardiac arrest, abnormal electrocardiogram (ECG), arrhythmia, or prolonged QT interval. Adverse event data were not provided by Health Catalyst or the VA.

### Analytic methods

Study follow-up began at slightly different time points within each group’s defined study design (S1 File). To improve baseline balance and minimize immortal time bias, in Aetion’s analyses, untreated were matched (on a number of key characteristics including calendar time and time since hospital admission) to treated patients on the day of first administration of HCQ (using risk set sampling). The index date for HCQ treated was the first day of treatment and the index date for the HCQ untreated was defined by the index date of the matched HCQ treated patient (for further detail see S1 File). For the VA, the index date was assigned to be 48 hours after hospital admission to avoid immortal time bias. For the other 4 datasets the index date was the date of admission.

Six groups used time-to-event analyses. The mortality outcome was primarily evaluated as in-hospital, for five of the groups, while the VA considered time to all-cause mortality within 30 days of the index date. Syapse examined the cumulative incidence of all-cause mortality during or after the index hospitalization. Health Catalyst conducted a primary analysis using mortality as time-to-event and sensitivity analysis treating mortality as binary.

### Statistical analysis

To examine the association between potential confounders and treatment with hydroxychloroquine and azithromycin, hydroxychloroquine alone, and azithromycin alone, we compared the distributions of each covariate in each treatment group and in the group receiving no treatment. In addition, we examined the distribution of adverse events across treatment groups.

Methods to assess the association between treatment and outcomes included logistic regression, competing risk analyses, and propensity score methods. Dascena employed Fine and Gray models for the subdistribution hazard ratio (HR) were used to examine the association between treatment and each of the outcome measures. This method allows for estimation of the incidence of events, despite the presence of a competing event that precludes the observation of the event of interest. Incidence was estimated using Breslow’s estimator. All individuals who had not experienced the event were censored at the end of the study period. Additional information about statistical methods used by each group can be found in the S1 File.

To adjust for baseline confounding variables, a subset of groups employed propensity score methods. Logistic regression was used by five groups to predict the probability of treatment with hydroxychloroquine, azithromycin, or both in the study population, conditional on all measured confounders. The VA estimated propensity scores using a gradient boosting machine, implemented using the packages ‘gbm’ and ‘WeightIt’ in R [13–15]. Aetion performed all analyses in the Aetion Evidence Platform v4.5. Propensity scores were then used to adjust for confounding either through inverse probability of treatment weighting (IPTW), propensity score matching, or adjustment on the propensity score. Health Catalyst conducted sensitivity analyses using unmatched, 1:1 matched, propensity score matched, propensity score adjusted, and propensity score binned techniques. Details of the adjustment methods used by each group are presented in Table 4 and in the S1 File.

**Table 4:** Key definitions and methodology.

Study Variable	Aetion	COTA	Dascena	Health Catalyst	Syapse	TriNetX	VA Dataset
**Inclusion Criteria**	Hospitalized patients defined as having any of the suspected COVID-19 criteria occurring in the 21 days prior to (and including) the hospital admission date (cohort entry date) OR in the discharge diagnosis	Hospitalized patients defined having positive SARS-CoV-2 diagnosis by RT-PCR	Hospitalized patients (defined as length of stay > 24 hours) defined as having a positive COVID-19 PCR test or diagnosis within five days of encounter	A discharge diagnosis of COVID 19 (ICD-10:U07.1) either primary or secondary to a specific list of other conditions	Hospitalized patients with malignant cancer (diagnosed in the last 5 years) at two health systems with confirmed COVID-19 diagnosis (via positive lab result and/or ICD code)	Hospitalized patients identified using coronavirus codes used in EMRs for COVID-19. Any Code must be present Jan. 20, 2020 or after to yield patients. Inpatient code required 2 weeks before or anytime after COVID-19	Hospitalized on or after first positive Sars-CoV-2 test. Treatment assignment groups were specified by the intent-to-treat design where HCQ/Azith/Both was/were initiated in the first 48-hours following admission. Comparison groups (Az, Neither) included individuals from hospitals where at least one person was prescribed HCQ.
**COVID-19 Definition**	Medical claim or chargemaster event with COVID-19-like diagnosis or Positive or presumptive positive viral lab test result	Positive SARS-CoV-2 diagnosis		ICD-10 diagnosis code		LOINC or ICD-10 diagnosis code	VA cases were based on the National Surveillance Tool classification following NLP and adjudication methodologies described(12)
**COVID-19 Diagnosis date**	Earliest date of confirmed COVID-19 recorded in 21 days pre-admission (inclusive) or admission date if diagnosis derived from discharge diagnosis	Date of confirmed COVID-19 diagnosis via PCR lab result		Earliest positive PCR lab collection data or clinical diagnosis of COVID-19	Date of confirmed COVID-19 diagnosis via ICD code or positive lab result	Minimum of positive PCR lab confirmed date or clinical diagnosis of COVID-19	All individuals included were required to have a case-defined diagnosis date prior to, or on the same day as, hospital admission.
					For patients with confirmation of COVID-19 diagnosis via ICD and lab test result, date of lab result is used		
**Index Date**	Initiation of treatment for HCQ+ patients; matched controls (HCQ-) were assigned an index date of their matched HCQ treated patient.	Date of hospital admission		Date of hospital admission		Date of hospital admission	Date of hospitalization + 48 hours
**Measure of Overall Health of Patients**	Mechanical Ventilation (used as a control outcome to refine comparative approach for future drug evaluation studies)	Mechanical Ventilation	Mechanical Ventilation	NA	Mechanical Ventilation	The rate of recovery, defined as an improvement from hospitalized with any oxygen support to either hospitalized on room air or discharge following the index date	Mechanical Ventilation
**Measure of Evidence of Benefit**	NA	Hospital discharge	Hospital discharge	NA	NA	The rate of recovery, defined as an improvement from hospitalized with any oxygen support to either hospitalized on room air or discharge following the index date	NA
**Adjusted Analysis Approach**	RSS+PS; Risk set sampling of HCQ untreated patients at time of HCQ administration in HCQ+, followed by propensity score matching based on key patient demographics and clinical characteristics	Propensity score matching and adjusting based on key patient demographics and clinical characteristics	Inverse probability weight adjusted	5-bin propensity stratified analysis	Not conducted. Only reported crude estimates	Inverse probability weight adjusted	Gradient boosted tree models for estimating weights to use in sIPTW with Cox proportional hazards

In each dataset, the association between each treatment (HCQ, HCQ+AZ) and each outcome was assessed in comparison to the non HCQ/AZ group (no treatment, “neither”). Therefore, treatment groups were not directly compared to each other in this analysis. For all analyses, a two-sided alpha of 0.05 without adjustment for multiple comparisons was used to determine statistical significance.

## Results

### Population

**Fig 3:**
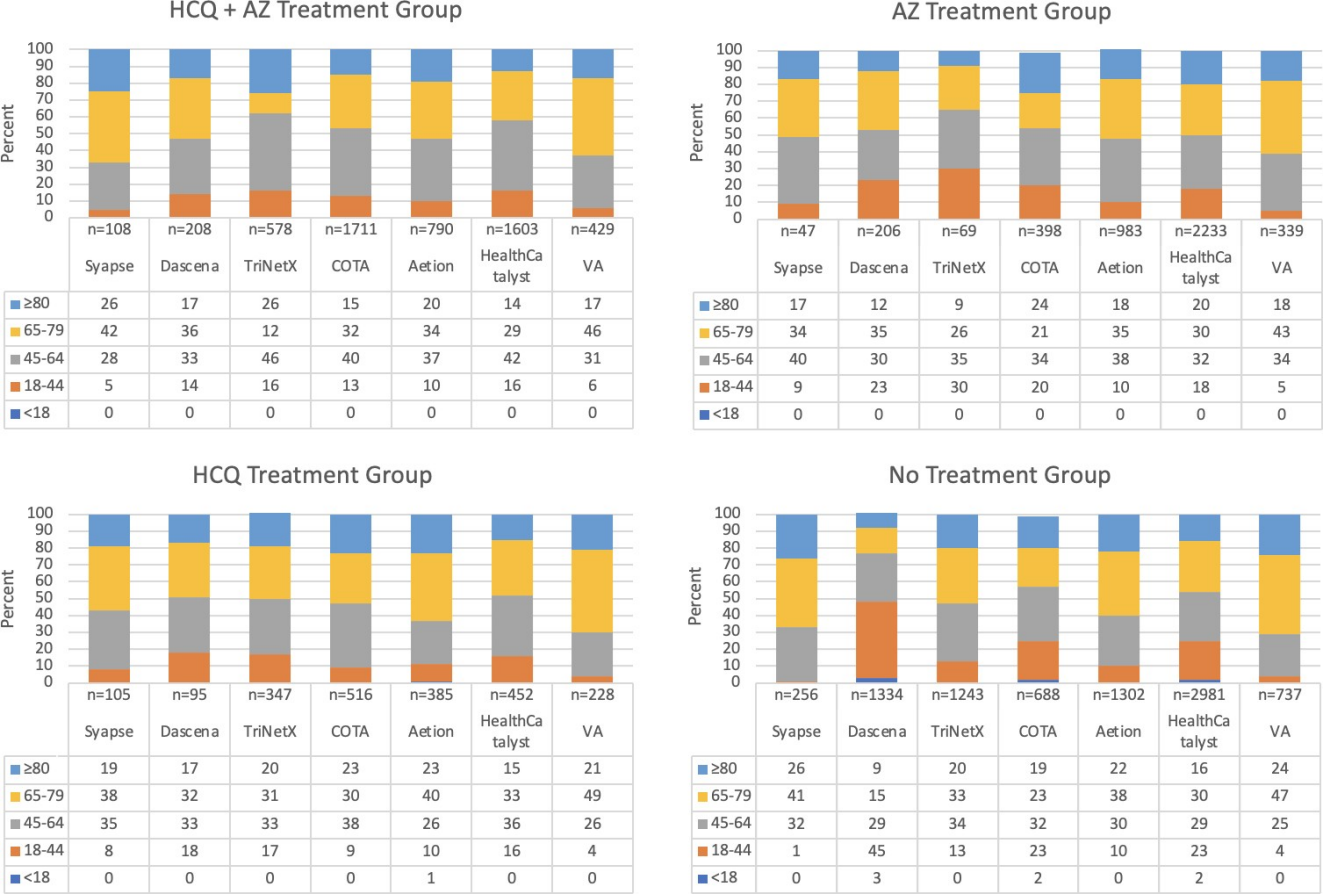
Age distribution by treatment group. Categorical age for each treatment group shows similarities and differences across data sets. Note. Numbers represent percent values.

**Fig 4:**
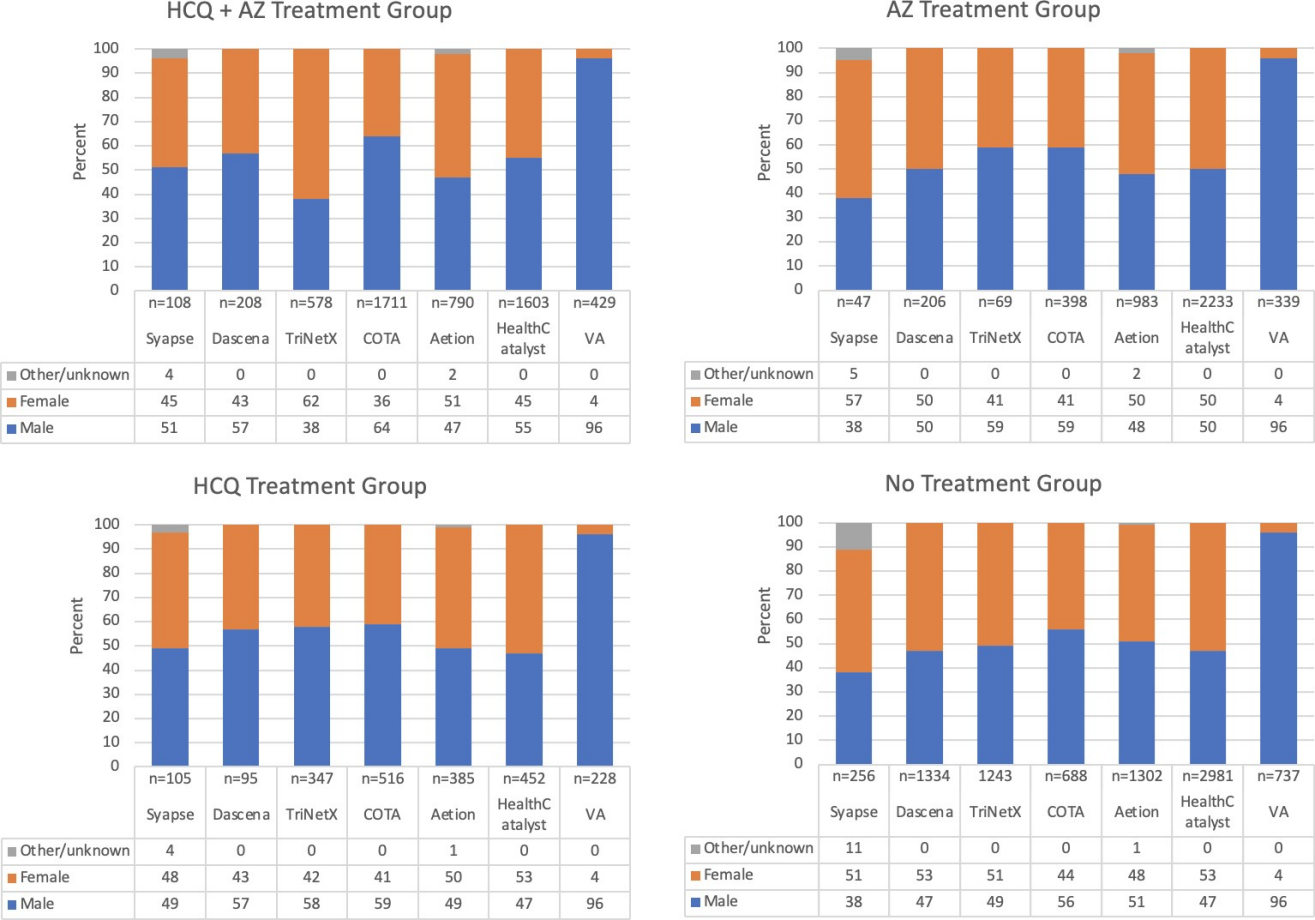
Sex distribution by treatment group. Sex distribution by treatment group shows similarities and differences across data sets. Note. Numbers represent percent values.

**Fig 5:**
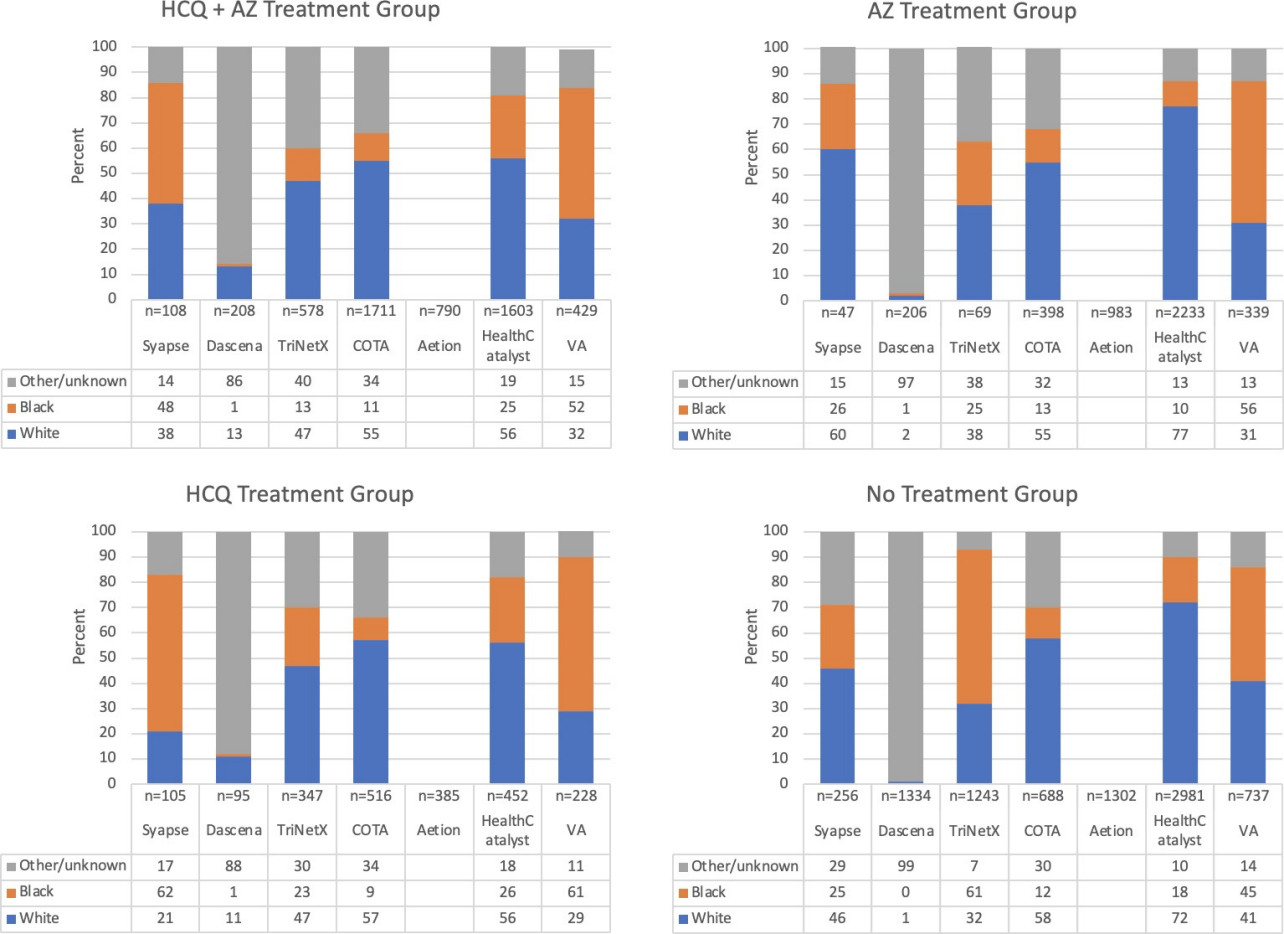
Race distribution by treatment group. Race distribution by treatment group shows similarities and differences across data sets. Note. Numbers represent percent values.

In total, 20,371 patient encounters from seven data sources were analyzed. Demographic characteristics of each dataset are laid out in Figs 3–5, with patient comorbidities presented in Table 5. Patient characteristics were similar across datasets. Patients receiving treatment with hydroxychloroquine were typically older than 45 with a larger proportion of males.

**Table 5:** Summary of comorbidities across groups.

**Hydroxychloroquine**	**Population Size, n**	**Any Cardiovascular Disease**	**Hypertension**	**Diabetes**	**Obesity**	**Coronary artery disease**	**Congestive heart failure**	**Chronic lung disease**
		**N (%)**	**N (%)**	**N (%)**	**N (%)**	**N (%)**	**N (%)**	**N (%)**
**Aetion**	385	244 (63)	172 (45)	118 (31)	79 (21)	42 (11)	54 (14)	57 (15)
**COTA/HMH**	516	334 (65)	313 (61)	185 (36)	186 (36)	94 (18)	Not assessed	37 (7)
**Dascena**	91	7 (8)	4 (4)	9 (10)	2 (2)	Not assessed	2 (2)	1 (1)
**Health Catalyst**	335	186 (56)	171 (51)	106 (32)	112 (33)	73 (22)	67 (20)	87 (26)
**Syapse**	105	49 (47)	29 (28)	37 (35)	79 (75)	9 (9)	8 (9)	0 (0)
**TriNetX**	347	194(56)	226(65)	135(39)	140(40)	133(38)	82(24)	Not assessed
**VA**	228	122 (54)	179 (79)	123 (54)	107 (47)	80 (35)	56 (25)	52 (23)
**Hydroxychloroquine + Azithromycin**	**Population Size, n**	**Any Cardiovascular Disease**	**Hypertension**	**Diabetes**	**Obesity**	**Coronary artery disease**	**Congestive heart failure**	**Chronic lung disease**
		**N (%)**	**N (%)**	**N (%)**	**N (%)**	**N (%)**	**N (%)**	**N (%)**
**Aetion**	790	438 (55)	271 (34)	197 (25)	177 (22)	54 (7)	63 (8)	84 (11)
**COTA/HMH**	1711	985 (58)	917 (54)	558 (33)	629 (37)	232 (14)	Not assessed	114 (7)
**Dascena**	206	13 (6)	11 (5)	23 (11)	12 (6)	Not assessed	2 (1)	14 (7)
**Health Catalyst**	1157	582 (50)	531 (46)	321 (28)	324 (28)	185 (16)	132 (11)	237 (21)
**Syapse**	108	70 (65)	48 (44)	38 (35)	92 (85)	13 (12)	10 (9)	1 (1)
**TriNetX**	578	225 (39)	280 (48)	202 (35)	198 (34)	129 (22)	53 (9)	Not assessed
**VA**	429	179 (42)	312 (73)	205 (48)	209 (49)	119 (28)	74 (17)	95 (22)
**Azithromycin**	**Population Size, n**	**Any Cardiovascular Disease**	**Hypertension**	**Diabetes**	**Obesity**	**Coronary artery disease**	**Congestive heart failure**	**Chronic lung disease**
		**N (%)**	**N (%)**	**N (%)**	**N (%)**	**N (%)**	**N (%)**	**N (%)**
**Aetion**	983	538 (55)	347 (35)	259 (26)	210 (21)	90 (9)	100 (10)	133 (14)
**COTA/HMH**	398	219 (55)	204 (51)	104 (26)	127 (32)	52 (13)	Not assessed	34 (9)
**Dascena**	201	28 (14)	22 (12)	20 (11)	7 (4)	Not assessed	7 (4)	7 (4)
**Health Catalyst**	1546	719 (47)	641 (42)	413 (27)	315 (20)	223 (22)	172 (11)	285 (18)
**Syapse**	47	30 (64)	21 (45)	13 (28)	43 (91)	8 (17)	8 (17)	2 (4)
**TriNetX**	69	35 (51)	37 (54)	24 (35)	24 (35)	17 (25)	11 (16)	Not assessed
**VA**	339	154 (45)	244 (72)	151 (45)	149 (44)	106 (31)	71 (21)	72 (21)
**Neither**	**Population Size, n**	**Any Cardiovascular Disease**	**Hypertension**	**Diabetes**	**Obesity**	**Coronary artery disease**	**Congestive heart failure**	**Chronic lung disease**
		**N (%)**	**N (%)**	**N (%)**	**N (%)**	**N (%)**	**N (%)**	**N (%)**
**Aetion**	1302	786 (60)	475 (37)	356 (27)	237 (18)	137 (11)	119 (9)	168 (13)
**COTA/HMH**	688	342 (50)	319 (46)	159 (23)	191 (28)	98 (14)	Not assessed	50 (7)
**Dascena**	1284	100 (17)	86 (15)	74 (13)	17 (3)	Not assessed	24 (2)	35 (6)
**Health Catalyst**	1101	611 (56)	556 (51)	337 (31)	283 (26)	233 (21)	225 (20)	276 (25)
**Syapse**	256	142 (55)	88 (34)	68 (27)	207 (81)	21 (8)	36 (14)	1 (0)
**TriNetX**	1243	646 (52)	764 (61)	453 (36)	378 (30)	248 (20)	245 (20)	Not assessed
**VA**	737	423 (57)	592 (80)	377 (51)	271 (37)	284 (39)	201 (27)	197 (27)

For the adverse event with most complete reporting, any arrhythmia, analysis did not support increased arrhythmia in patients receiving hydroxychloroquine versus those not (Fisher’s Exact Test p-value 0.462) (Table 6).

**Table 6:** Frequency of adverse events across each treatment group.

**Hydroxychloroquine**	**Population Size, n**	**Any arrhythmia**	**Diarrhea**	**MI, Stroke, CABG/PCI**	**Any conduction disorder**	**hypoglycemia**
		**N (%)**	**N (%)**	**N (%)**	**N (%)**	**N (%)**
Aetion*	385	92 (24)	15 (4)	29 (8)	13 (3)	12 (3)
**COTA/HMH**	516	36 (7)	0 (0)	0 (0)	12 (2)	0 (0)
**Dascena**	91	1 (1)	0 (0)	0 (0)	0 (0)	0 (0)
**Syapse**	105	7 (7)	1 (1)	4 (4)	0 (0)	1 (1)
**TriNetX**	347	2 (1)	43 (12)	3 (1)	35 (10)	3 (1)
**Hydroxychloroquine + Azithromycin**	**Population Size, n**	**Any arrhythmia**	**Diarrhea**	**MI, Stroke, CABG/PCI**	**Any conduction disorder**	**hypoglycemia**
		**N (%)**	**N (%)**	**N (%)**	**N (%)**	**N (%)**
Aetion*	790	193 (24)	50 (6)	49 (6)	23 (3)	29 (4)
**COTA/HMH**	1711	88 (5)	0 (0)	0 (0)	35 (2)	0 (0)
**Dascena**	206	10 (5)	3 (2)	0 (0)	0 (0)	3 (2)
**Syapse**	108	15 (14)	4 (4)	4 (4)	0 (0)	0 (0)
**TriNetX**	578	4 (1)	96 (17)	9 (2)	54 (9)	2 (0)
**Azithromycin**	**Population Size, n**	**Any arrhythmia**	**Diarrhea**	**MI, Stroke, CABG/PCI**	**Any conduction disorder**	**hypoglycemia**
		**N (%)**	**N (%)**	**N (%)**	**N (%)**	**N (%)**
Aetion*	983	232 (24)	51 (5)	53 (5)	36 (4)	36 (4)
**COTA/HMH**	398	11 (3)	0 (0)	0 (0)	Not assessed	0 (0)
**Dascena**	201	3 (2)	2 (1)	0 (0)	0 (0)	0 (0)
**Syapse**	47	6 (13)	3 (6)	3 (6)	1 (2)	0 (0)
**TriNetX**	69	0 (0)	0 (0)	0 (0)	0 (0)	0 (0)
**Neither**	**Population Size, n**	**Any arrhythmia**	**Diarrhea**	**MI, Stroke, CABG/PCI**	**Any conduction disorder**	**hypoglycemia**
		**N (%)**	**N (%)**	**N (%)**	**N (%)**	**N (%)**
Aetion*	1302	325 (25)	67 (5)	86 (7)	85 (7)	80 (6)
**COTA/HMH**	688	21 (3)	0 (0)	0 (0)	Not assessed	0 (0)
**Dascena**	1284	5 (1)	1 (0)	0 (0)	0 (0)	3 (1)
**Syapse**	256	30 (12)	3 (1)	8 (3)	3 (1)	2 (1)
**TriNetX**	1243	24 (2)	35 (3)	9 (1)	149 (12)	2 (0)

*Adverse event data from discharge diagnoses.

### Outcomes

Frequencies of each evaluated outcome are displayed in Table 7. In the Syapse study population of patients with cancer and COVID-19, crude all-cause mortality estimates were greatest among patients receiving hydroxychloroquine plus azithromycin (29.6%), followed by azithromycin alone (21.3%). The crude cumulative incidence of mechanical ventilation was also greatest in the hydroxychloroquine plus azithromycin treatment arm (35.2%), followed by hydroxychloroquine alone (22.9%).

**Table 7:** Frequencies of outcome for each treatment group.

**No Treatment**	**Mortality**	**Mechanical Ventilation**	**Evidence of Benefit**
Aetion* (n = 1302)	Not assessed	95 (7%)	Not assessed
COTA/HMH (n = 688)	123 (18%)	44 (6%)	492 (72%)
Dascena (n = 1334)	47 (4%)	44 (3%)	539 (40%)
Health Catalyst (n = 1101)	203 (18%)	Not assessed	Not assessed
Syapse (n = 256)	33 (12.9%)	16 (6.2%)	Not assessed
TriNetX (n = 1243)	188 (15%)	Not assessed	728 (59%)
VA (n = 737)	141 (19%)	69 (9%)	Not assessed
**Hydroxychloroquine**	**Mortality**	**Mechanical Ventilation**	**Evidence of Benefit**
Aetion* (n = 385)	Not assessed	48 (12%)	Not assessed
COTA/HMH (n = 516)	154 (30%)	111 (22%)	270 (52%)
Dascena (n = 95)	20 (21%)	14 (15%)	65 (68%)
Health Catalyst (n = 335)	50 (15%)	Not assessed	Not assessed
Syapse (n = 105)	18 (17%)	24 (23%)	Not assessed
TriNetX (n = 347)	45 (13%)	Not assessed	176 (46%)
VA (n = 228)	49 (21%)	32 (14%)	Not assessed
**Hydroxychloroquine + Azithromycin**	**Mortality**	**Mechanical Ventilation**	**Evidence of Benefit**
Aetion* (n = 790)	Not assessed	128 (16%)	Not assessed
COTA/HMH (n = 1711)	428 (25%)	479 (29%)	1,089 (64%)
Dascena (n = 208)	46 (29%)	73 (35%)	124 (60%)
Health Catalyst (n = 1157)	212 (18%)	Not assessed	Not assessed
Syapse (n = 108)	32 (30%)	38 (35%)	Not assessed
TriNetX (n = 578)	66 (11%)	Not assessed	316 (55%)
VA (n = 429)	90 (21%)	64 (15%)	Not assessed
**Azithromycin**	**Mortality**	**Mechanical Ventilation**	**Evidence of Benefit**
Aetion* (n = 983)	Not assessed	144 (15%)	Not assessed
COTA/HMH (n = 398)	97 (24%)	43 (11%)	266 (67%)
Dascena (n = 206)	8 (4%)	28 (14%)	96 (47%)
Health Catalyst (n = 1546)	280 (18%)	Not assessed	Not assessed
Syapse (n = 47)	10 (21%)	3 (6%)	Not assessed
TriNetX (n = 69)	8 (12%)	Not assessed	35 (51%)
VA (n = 339)	56 (17%)	39 (12%)	Not assessed

*To align with other Parallel Analysis partners, Aetion assessed the risk of incident mechanical ventilation among patients in the risk set sampled population, before propensity score matching (used for T1-6). Outcome frequencies in T6 are reported among patients without record of ventilation prior to or concurrent with treatment index.

Given constraints of the data, not all groups performed an adjusted analysis. Six groups (Dascena, Health Catalyst, TriNetX, Aetion, COTA/HMH and the VA) conducted adjusted analyses. Among the 3 groups that conducted comparative analyses between hydroxychloroquine plus azithromycin and monotherapy treatment groups (Dascena, Health Catalyst and the VA), after adjusting for confounding, hydroxychloroquine alone was not found to be associated with mortality, overall patient condition, or benefit to the patient. Interrogation of confounding mitigation demonstrated an ability to balance across treatment groups especially on key characteristics such as age and comorbid burden. Hydroxychloroquine plus azithromycin was similarly not associated with any of the outcomes assessed in this study. Adjusted results are presented in Table 8.

**Table 8:** Adjusted hazard ratios for each treatment group.

Hydroxychloroquine	Mortality	Mechanical Ventilation	Evidence of Benefit
Aetion*	Not assessed	1.29 (0.96, 1.74)	Not assessed
COTA/HMH	1.22 (0.93, 1.60)	Not assessed	Not assessed
Dascena	2.6 (0.82, 8.0)	0.32 (0.04, 2.4)	0.80 (0.37, 1.7)
Health Catalyst	Not assessed	Not assessed	Not assessed
TriNetX	1.4 (0.97, 2.05)	Not assessed	1.03 (0.86, 1.23)
VA	1.18 (0.81, 1.72)	1.31 (0.81, 2.13)	Not assessed
**Hydroxychloroquine + Azithromycin**	**Mortality**	**Mechanical Ventilation**	**Evidence of Benefit**
Aetion	Not assessed	Not assessed	Not assessed
COTA/HMH	1.16 (0.90, 1.51)	Not assessed	Not assessed
Dascena	1.9 (0.91, 4.1)	2.5 (1.2, 5.2)	0.90 (0.58, 1.4)
Health Catalyst	1.09 (0.76, 1.56)	Not assessed	Not assessed
TriNetX	0.99 (0.73, 1.35)	Not assessed	0.87 (0.75, 1.01)
VA	1.18 (0.88, 1.58)	1.54 (1.07, 2.23)	Not assessed
**Azithromycin**	**Mortality**	**Mechanical Ventilation**	**Evidence of Benefit**
Aetion	Not assessed	Not assessed	Not assessed
COTA/HMH	1.31 (0.95, 1.82)	Not assessed	Not assessed
Dascena	0.79 (0.31, 1.6)	1.7 (1.0, 3.0)	1.4(1.0, 1.8)
Health Catalyst	Not assessed	Not assessed	Not assessed
TriNetX	Not assessed	Not assessed	Not assessed
VA	0.89 (0.63, 1.25)	1.03 (0.66, 1.61)	Not assessed

*Aetion reports adjusted hazard ratios for incident mechanical ventilation events among hydroxychloroquine initiators and untreated controls matched with both risk set sampling and propensity score models. Hazard ratios are derived from all hydroxychloroquine initiators and all matched controls, regardless of azithromycin treatment.

Based on these results, hydroxychloroquine and azithromycin, alone or in combination, did not appear to impact outcomes among COVID-19 patients. These results were consistent across datasets, with the only notable difference being the evidence of benefit with azithromycin treatment observed by Dascena. Besides the primary analysis, Health Catalyst performed additional sensitivity analyses based upon (a) inclusion criteria (positive lab or ICD-10; require primary discharge ICD-10; allow treatment initiation after the second day), (b) treating mortality as binary, (c) different confounders and confounding mitigation techniques. Results were as expected: (a) broader inclusion criteria resulted in more baseline group differences, (b) treating mortality as binary reduced potential treatment benefit especially when not requiring rapid treatment initiation (e.g., immortal time bias), and (c) more extreme results were observed in the absence of any confounder adjustment technique and different techniques had small impacts of results.

## Discussion

In this study, a consortium of groups conducted parallel analyses of the effects of hydroxychloroquine, azithromycin, and their combination on health outcomes of COVID-19 patients. By conducting parallel analyses that aligned on a common protocol, while allowing for flexibility within each group to define covariates, exposure and outcome identification, this study aimed to provide a robust description of outcomes associated with the use of hydroxychloroquine for the treatment of COVID-19.

Among five sites that contributed race data, Syapse, the VA and Health Catalyst reported that Black patients made up a larger distribution of HCQ/HCQ+AZM recipients compared to their distribution without these treatments. Race did not appear to be associated with HCQ administration in the COTA/HMH dataset; and White patients represented a greater proportion of HCQ/HCQ+AZM recipients in the TriNetX dataset as compared to their distribution among those without HCQ treatment.

Across all datasets and treatment groups, the most prominent pre-existing conditions tended to be any cardiovascular disease, hypertension, diabetes and obesity. Overall, obesity was more prevalent among the HCQ treatment groups than in the neither group.

For most data partners, the proportion of patients treated with any of these comorbidities was lower or no different in the HCQ groups than in the neither group–with the exception of Syapse, which was a cancer cohort.

There are several limitations to this study that must be acknowledged. First, despite our goal of carrying out the same set of analyses on multiple datasets, analyses could not be carried out identically on all datasets due to differences in and limitations of the data. In particular, not all groups were able to carry out an adjusted analysis that controlled for confounding variables. Second, due to stratification on treatment groups, some analyses were conducted on small sample sizes. Data was also limited to those collected from United States sources. These results may therefore not be generalizable to international settings. Coding of certain outcomes, particularly adverse events, may have been incomplete. Therefore, not all outcomes may have been captured, potentially limiting the accuracy of our results.

These analyses were conducted in parallel among 7 individual groups using their own datasets. Characteristics, definitions, and methodologies used by each of the groups are summarized in Tables 1 and 4. A goal of this project was to develop a common analytical plan for multiple groups to apply to different datasets as they continued to aggregate data on the experience of diagnosis, treatment, and outcomes associated with COVID-19. Given the novelty of the virus and magnitude of the pandemic, the use of data derived from various sources of healthcare data presents an opportunity to augment clinical trial data with information about patients not enrolled in clinical studies, and provide information about treatment patterns and observations about those experiences in large, diverse populations. By using a common analysis plan, the resulting observations can be more readily compared and if consistent, can further support the findings among individual studies.

In this instance, several of the studies were already underway when the parallel analysis was started. This made it difficult to align on all aspects of the analyses and data parameters. As future parallel analyses are considered, it is important that participants seek to develop as much uniformity to the definitions and methods as possible. However, given the different sources of data, some aspects of the analysis will need to be tailored to the individual dataset and those variations should be clearly described. As future study questions are developed to further characterize COVID-19 treatments, it is important to select the sources of data that are best fit to answer each specific question. In addition, future parallel analysis should consider using a stepwise approach to perform a sample size, demographic, and feasibility assessment and use that initial step to optimally design subsequent comparative analyses.

## Conclusion

The Evidence Accelerator successfully brought together seven partners to execute analyses in disparate populations. Representing more than 20,000 patients with COVID-19 across the U.S, we found similar trends in those getting HCQ treatment–despite minor differences in coding and cohort entry. Across the 5 groups who ran comparative analyses, we observed no association between HCQ treatment and mortality, overall patient condition, or evidence of benefit.

## Supporting information

S1 File (DOCX)
