# Supplementary material for: COVID-19 Evidence Accelerator: A parallel analysis to describe the use of Hydroxychloroquine with or without Azithromycin among hospitalized COVID-19 patients
Source: PLoS One. 2021 Mar 17;16(3):e0248128. doi: 10.1371/journal.pone.0248128 (PMC7968637; doi:10.1371/journal.pone.0248128)
Supplement: S1 File — (DOCX) [file pone.0248128.s001.docx]

**S1 File:**

**Comorbid diagnoses considered to be confounders by each group:**

**Aetion:** See *Aetion Fig 1* for a full list of confounders included in final risk set sampling (RSS) and propensity score (PS) models.

**COTA/Hackensack Meridian Health:** diabetes, chronic obstructive pulmonary disease (COPD), hypertension, cancer, coronary artery disease, stroke, heart failure, arrhythmia, renal failure, and number of comorbidities at baseline.

**Dascena:** Comorbidities and acute diagnoses included sepsis, cardiovascular disease, renal disease, congestive heart failure, peripheral vascular disease, cerebral vascular disease, chronic pulmonary disease, mild liver disease, severe liver disease, diabetes with complication, diabetes without complication, rheumatologic disease, myocardial infarction, hypertension, organ transplant, human immunodeficiency virus (HIV), cancer, psychiatric diagnosis, alcohol abuse, pneumonia, chronic obstructive pulmonary disease (COPD), dementia and obesity.

**Health Catalyst:** Comorbidities and acute diagnoses included sepsis, cardiovascular disease, renal disease, congestive heart failure, peripheral vascular disease, cerebral vascular disease, chronic pulmonary disease, mild liver disease, severe liver disease, diabetes with complication, diabetes without complication, rheumatologic disease, myocardial infarction, hypertension, cancer, alcohol abuse, pneumonia, chronic obstructive pulmonary disease (COPD), and obesity.

**TriNetX:** age, race, oxygen status at baseline, gender, chronic pulmonary disease, diabetes, hypertension, asthma, rheumatic arthritis, HIV status, obesity, cardiovascular disease, coronary artery disease, congestive heart disease, cancer, chronic liver disease, pulmonary arterial disease, chronic kidney disease, use of ARB or ACE inhibitor, cerebrovascular disease, and insulin use.

**Veterans Affairs:** Acute measures include vitals (blood pressure, oxygen, pulse, respiratory rate, temperature, body mass index), selected labs (c-reactive protein, ferritin, d-dimer, lactate dehydrogenase, alanine transferase, aspartate aminotransferase, estimated glomerular filtration rate, white blood cell count, and platelet count), use of inpatient HCQ contraindications, use of inpatient alternative COVID treatments (lopinavir/ritonavir, remdesivir, dexamethasone, methylprednisolone, or tocilizumab), calendar week of admission and VA station. Baseline conditions included one inpatient or two outpatient visits with diagnosis codes (ICD-10) for: acute myocardial infarction (AMI), cardiomyopathy (CARD), congestive heart disease (CHD), heart failure (HF), cerebrovascular accident (CVA), peripheral vascular disease (PVD), hypertension, chronic kidney disease, cancer, severe liver disease, asthma, chronic obstructive pulmonary disease (COPD), bronchitis, alcohol use disorder, diabetes, dementia, rheumatoid arthritis, venous thromboembolism, ischemic stroke, arterial disease, lupus, multiple sclerosis, and a frailty score.^1^ Baseline labs included: Blood urea nitrogen, high density lipoprotein cholesterol (HDL-C), low density lipoprotein cholesterol (LDL-C), total cholesterol count, triglycerides, hemoglobin, sodium, and neutrophils and lymphocytes count. Chronic medication use included outpatient fills for angiotensin receptor blockers (ARBs), angiotensin converting enzyme (ACE) inhibitors, steroids, antiplatelet therapy, and anticoagulants. Additional measures collected over time were AUDIT-C, smoking status, and demographics (race, ethnicity, admission from a long-term care facility, urbanicity, and geographic region).

1. Orkaby, A. R. *et al.* The burden of frailty among US veterans and its association with mortality, 2002–2012. *Journals Gerontol. Ser. A* **74**, 1257–1264 (2019).

**Additional information about analyses performed by individual groups:**

**Aetion adjusted analyses:**

As an extension of descriptive analyses on hydroxychloroquine treatment patterns and clinical characteristics, Aetion implemented a limited set of adjusted analyses with risk set sampling and propensity score matching techniques to reduce/avoid bias. Assuming HCQ treatment among patients with COVID-19 does not cause in-hospital mechanical ventilation, the mechanical ventilation composite endpoint was used as a control outcome in order to refine the comparative approach for future drug evaluation studies.

Aetion used an incident user cohort design to assess the risk of mechanical ventilation among hospitalized COVID-19 patients treated in-hospital with hydroxychloroquine as compared to matched controls, irrespective of azithromycin treatment. Using risk set sampling, new users of hydroxychloroquine were matched to controls randomly selected among patients not treated with hydroxychloroquine as of the treated patient's index date, with direct matching (1:2 fixed ratio) on calendar date of treatment index (+/-3 days), age (+/-3 yrs), gender, and number of days since hospital admission. For adjusted analyses, the risk set sampled hydroxychloroquine treated and untreated populations were further matched on propensity scores estimated using logistic regression with demographic and clinical risk factors, including covariates related to baseline medical history, admitting status, and disease severity at treatment (see study diagram for list of confounders). Note patients with any record of hydroxychloroquine use or ventilation procedures during the 90 days prior to treatment index (inclusive) were excluded from the adjusted analysis (**Aetion Figure 1**).

Balance between hydroxychloroquine initiators and matched controls was evaluated by comparison of standardized differences in covariates between treatment groups. All standardized differences were less than 0.2 after risk set sampling and propensity score matching, indicating good balance between treatment groups (**Aetion Figure 2**).

The primary analysis was an as-treated design, with follow-up for mechanical ventilation events beginning 1 day after treatment index and ending on the hospital discharge date. Patients matched as untreated controls who later initiated hydroxychloroquine were censored at crossover.

**Aetion Figure 1. Cohort study design, assessment windows, and confounders**

*
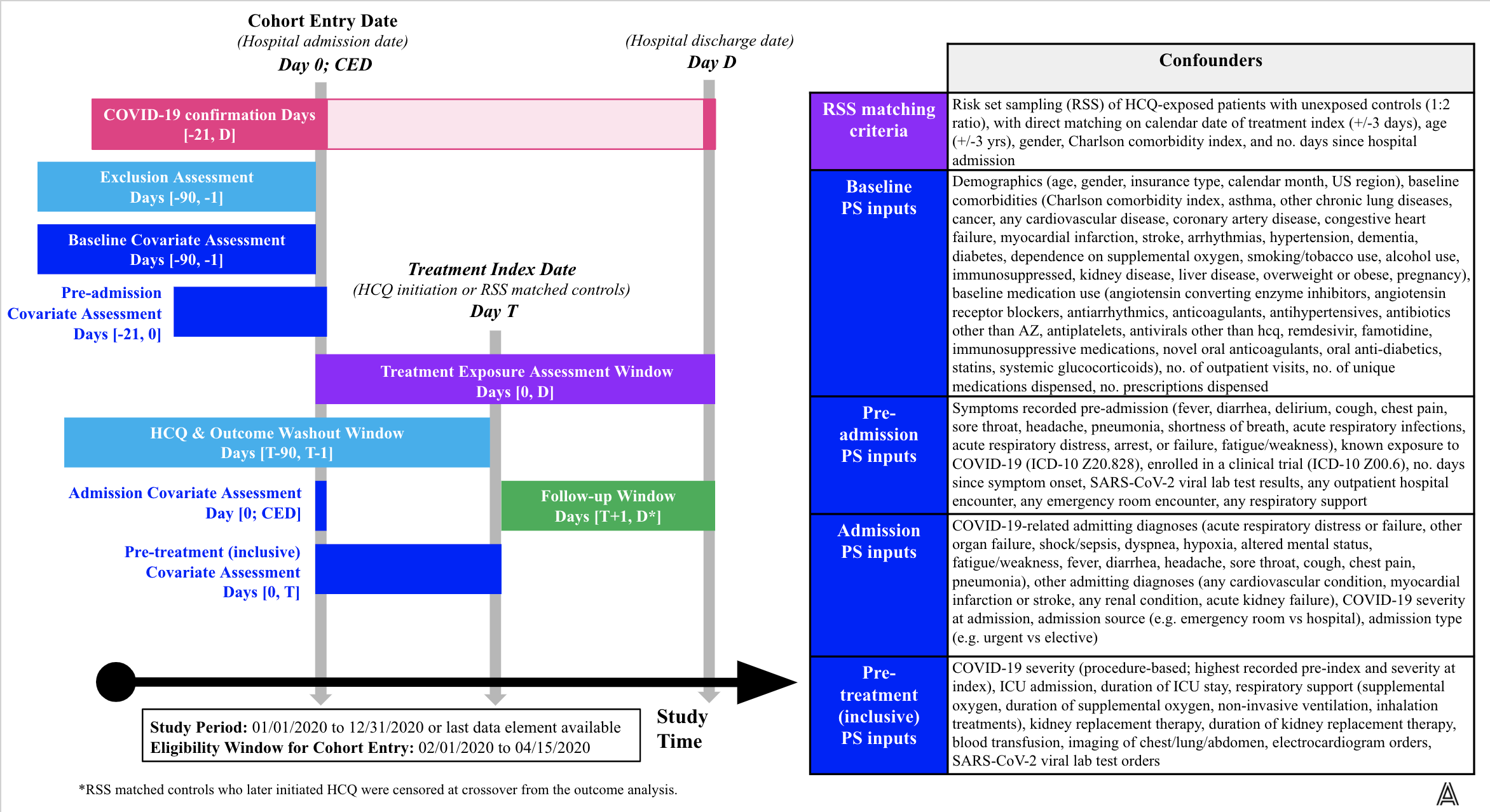
*

**Aetion Figure 2. Average Standardized Differences**


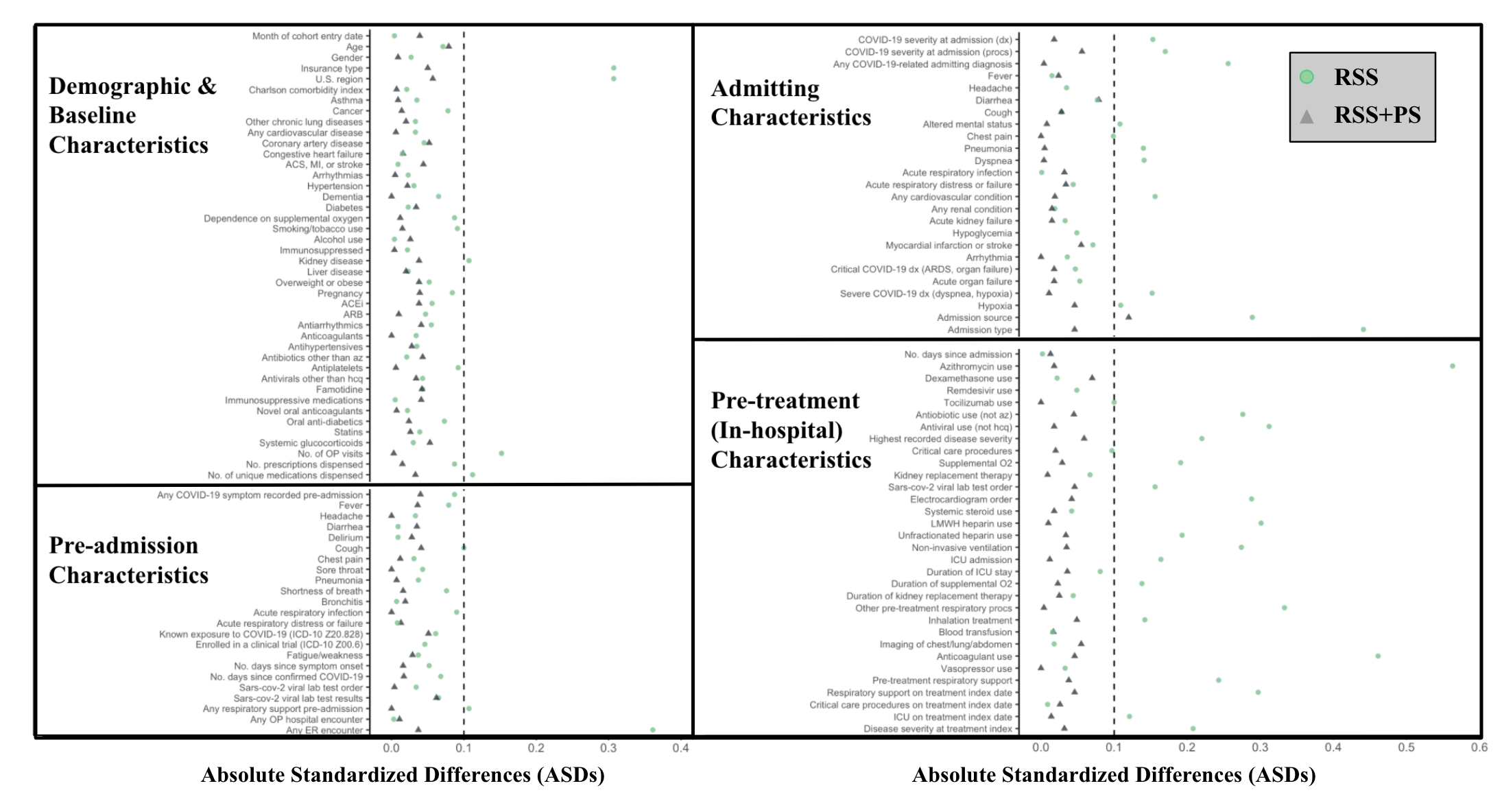


**COTA/HMH adjusted analyses:**

COTA partnered with Hackensack Meridian Health System (HMH) to complete an analysis investigating the effectiveness of hydroxychloroquine (HCQ) either alone or in combination with azithromycin to treat COVID-19 infection. The analysis utilized the HMH Real-world Evidence COVid-RegistrY (RE-COV-RY), a retrospective, de-identified database comprised of EHR-derived data from hospitalized patients treated at an HMH facility in New Jersey. Data abstraction was conducted by research nurses and physicians from the John Theurer Cancer Center at Hackensack University Medical Center using REDCap (Research Electronic Data Capture). The analysis was completed using the statistical analysis plan developed by the COVID-19 Evidence Accelerator (version 5/20/2020).

Patients were included in the analysis if they had a SARS-CoV-2 diagnosis by reverse-transcriptase polymerase chain reaction (RT-PR), or a presumptive positive clinical diagnosis, and were hospitalized between March 28, 2020 and the end of study period, May 29^th^, 2020. Index date for analyses was the date of hospital admission. Comorbidities were defined as being diagnosed prior to hospitalization for COVID-19. Exposure to hydroxychloroquine and/or azithromycin treatment was defined as any exposure during the hospitalization. Adverse events were identified as occurring any time after index date of hospital admission. Demographic characteristics, comorbidities and adverse events were summarized across treatment groups using descriptive statistics. To complete the adjusted mortality analysis, logistic regression was used to predict the probability of treatment with hydroxychloroquine, azithromycin, or both in our study population, conditional on demographic factors, comorbidities, and presenting vitals. Variables included in the final adjusted models included age, sex, race, nursing home status, practice setting, smoking history, diabetes, insulin use, chronic obstructive pulmonary disease (COPD), hypertension, cancer, coronary artery disease, stroke, heart failure, arrythmia, renal failure, number of comorbidities at baseline, fever, respiratory rate, oxygenation status, C-reactive protein (CRP) level, quick Sepsis-related Organ Failure Assessment (qSOFA) score, and ICU status. Probability of each treatment was calculated separately. Lasso regression using five-fold cross validation was utilized for variable selection for the logistic models, with prioritization given to variables that were significant in determining the outcome of interest. Two-sided alpha of 0.05 was used to determine statistical significance. All individuals who had not experienced the event were censored at the end of the study period (last data collection date), including individuals who were discharged from the hospital.

Propensity scores were then used to adjust for confounding either through inverse probability of treatment weighting (IPTW), propensity score matching, or adjustment on the propensity score. To account for outliers in the inverse probability of treatment weighting model, the sample population was restricted to patients with overlapping propensity scores between the two groups (treated vs. untreated) and further restricted to patients with a propensity score in the 98th percentile. For propensity score matching, scores were matched using nearest-neighbors in a 1:1 ratio between treated and untreated patients. Matching with and without replacement was attempted, varying the caliper size from 0.1 to 0.2. Adjusting for the propensity score included dividing the cohort into quintiles and including four of the quintiles in the final Cox model as covariates, with the remaining quintile being included in the intercept. Post-matching showed balance improvement with mean difference <0.1 with the exception of age (numeric) which had mean differences from 0.5 to 2.3 years.

A total of 3,313 patients meeting the inclusion criteria were included in the final analysis. Demographic characteristics of the population are summarized in Table 1. We observed that patients who received HCQ alone or in combination with azithromycin had greater proportions of comorbidities including any cardiovascular disease, hypertension, obesity, and kidney disease (Table 2). These findings indicate a generally sicker patient population and highlight the need for adequate adjustment for potential confounding variables. Additionally, higher proportions of patients receiving HCQ experienced arrhythmia, shock or renal failure in the hospital as compared to patients who received azithromycin alone or neither drug (Table 3). In our primary analysis, we observed a non-significant greater risk of mortality among patients treated with HCQ alone (_adj_HR = 1.22, 95% CI: 0.93 - 1.60) and in combination with azithromycin (_adj_HR = 1.16, 95% CI: 0.90 - 1.51). Although these results did not reach statistical significance, the directionality was consistent across multiple methods of propensity score adjustment and with our previously published findings which found no statistically significant benefit of HCQ administration.

**TriNetX Adjusted Analyses:**

TriNetX, a global health research network, was used to create a de-identified dataset of electronic medical records (diagnoses, procedures, medications, laboratory values, genomic information) for patients with COVID-19. The data is de-identified based on standard defined in Section §164.514(a) of the HIPAA Privacy Rule. The process by which Data Sets are de-identified is attested to through a formal determination by a qualified expert as defined in Section §164.514(b)(1) of the HIPAA Privacy Rule.

Among a set of US-based health care organizations, 21,841 patients with least one COVID-19-related diagnosis (U07.1, B97.29, B34.2) or positive SARS coronavirus 2 RNA test between Jan 20, 2020 and April 27, 2020 were identified. Patients were excluded if they participated in a clinical research program after Jan 20, 2020 or if they had no record of an inpatient stay in the two-weeks before or after their COVID-19 diagnosis.

Patients were stratified by hydroxychloroquine (HCQ), HCQ and Azithromycin, or no HCQ treatment at any time during their hospitalization. For each treatment group, mortality and the rate of recovery were assessed. The rate of recovery was defined as an improvement from hospitalization with any oxygen support to either hospitalized on room air or discharge at day 28 following the index date. The index date was defined as the first day of the hospitalization.

The following variables were used to adjust for confounding: age, race, oxygen status at baseline, gender, chronic pulmonary disease, diabetes, hypertension, asthma, rheumatic arthritis, HIV status, obesity, cardiovascular disease, coronary artery disease, congestive heart disease, cancer, chronic liver disease, pulmonary arterial disease, chronic kidney disease, use of ARB or ACE inhibitor, cerebrovascular disease, and insulin use.

Hazard ratios describe the association between treatment and outcomes using the no HCQ as the reference and adjustment conducted using inverse probability of treatment weights (IPTW).

**Dascena Adjusted Analysis:**

Dascena included patients diagnosed with COVID-19 at one of 6 community hospitals. COVID-19 was identified through a positive polymerase chain reaction (PCR) test or the presence of a COVID-19-related ICD discharge code (U07.1, B97.21, B97.29, J12.81, or B34.2). Dascena required the positive PCR test occur on the same day as the index hospitalization to ensure that patients were experiencing active COVID-19 infection during their hospital stay. Dascena enrolled patients between March 10 and June 4, 2020. To maximize the study sample, no patients with a positive COVID-19 diagnosis were excluded.

Dascena considered patients to be treated with hydroxychloroquine, azithromycin, or their combination if they received any of the above treatments at any point during their hospital stay. Initiation of hydroxychloroquine and azithromycin were not required to occur on the same day (**Dascena Figure 1**).

To control for confounding, Dascena collected information on the following comorbidities: sepsis, cardiovascular disease, renal disease, congestive heart failure, peripheral vascular disease, cerebral vascular disease, chronic pulmonary disease, mild liver disease, severe liver disease, diabetes with complication, diabetes without complication, rheumatologic disease, myocardial infarction, hypertension, organ transplant, human immunodeficiency virus (HIV), cancer, psychiatric diagnosis, alcohol abuse, pneumonia, chronic obstructive pulmonary disease (COPD), dementia and obesity. Adjustment was also made for admission lab values, concurrent medication usage, and demographic information including age, sex, race/ethnicity, and income.

Time-to-event models were adjusted solely through the use of inverse probability of treatment (IPTW) weights. Logistic regression models, adjusted for the covariates listed above, were used to predict the probability of treatment with hydroxychloroquine, azithromycin, or their combination. Separate models were built for each treatment group.

For both in-hospital mortality and mechanical ventilation outcomes, separate models were built comparing each treatment group to the group receiving neither hydroxychloroquine nor azithromycin, with each patient weighted by the appropriate inverse probability of treatment weight. In-hospital mortality was identified through discharge disposition, and mechanical ventilation was identified through ICD-10 and CPT codes.

**Dascena Figure 1. Study Diagram**


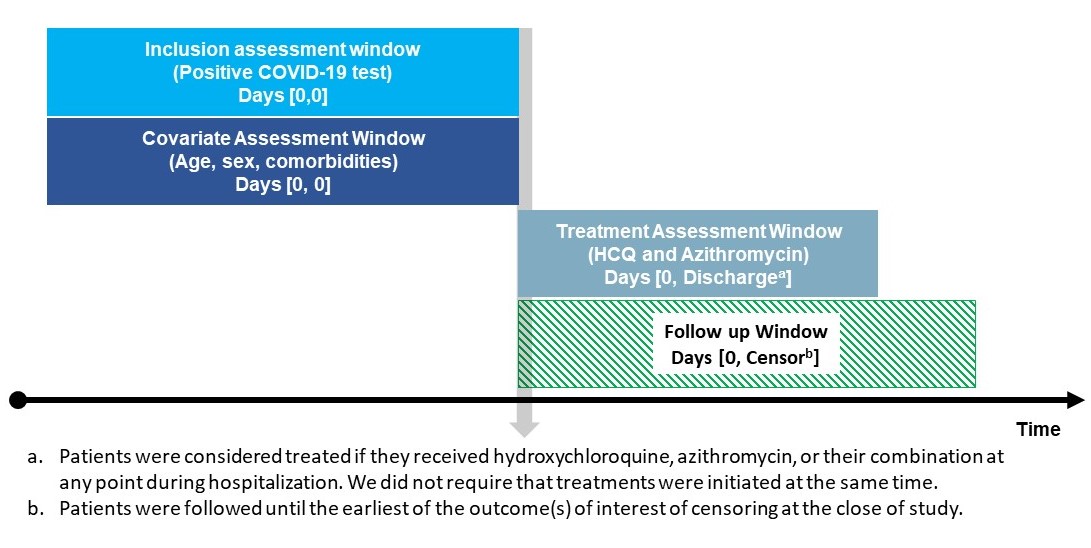


**Syapse:**

**Syapse Figure 1. Cohort study design and timeframes**


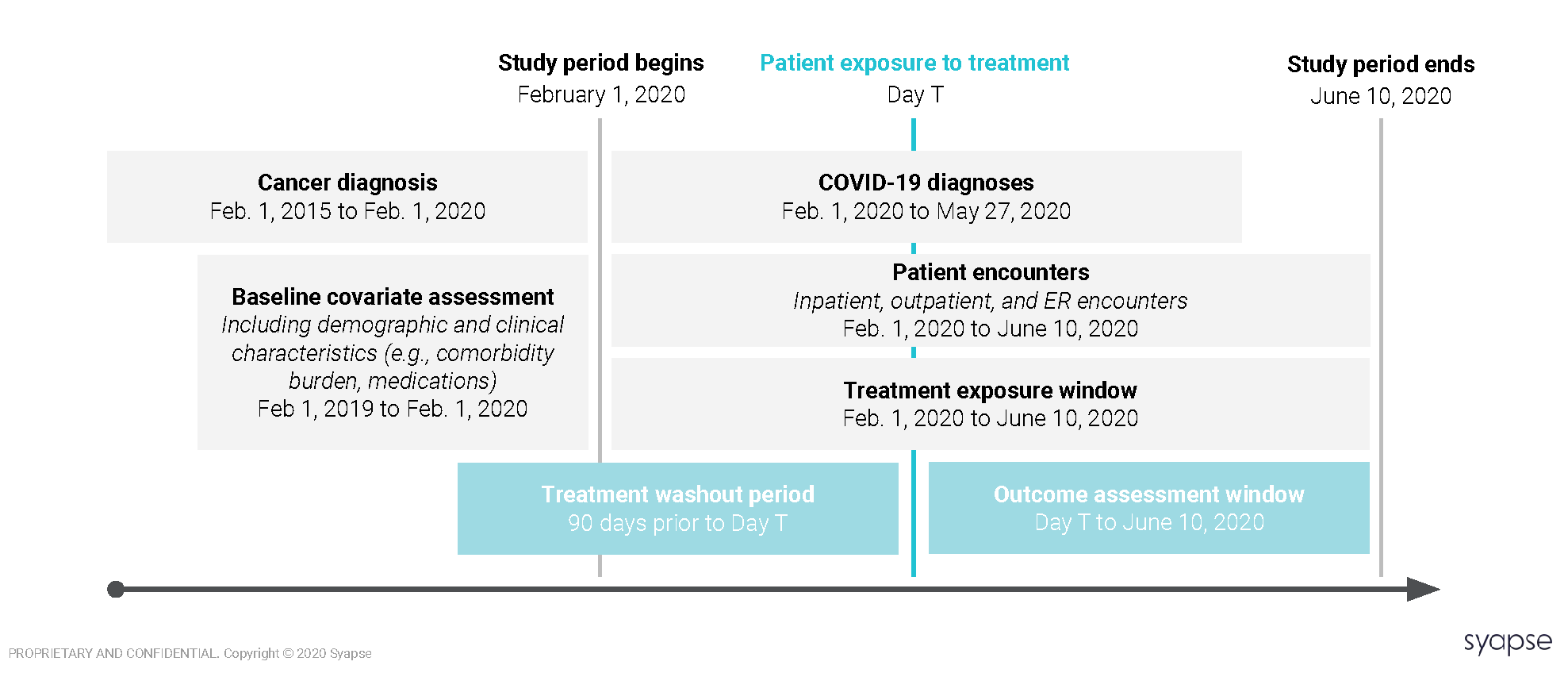


**VA Adjusted Analyses:**

The goal of the Veteran’s Health Administration (VA) Hydroxychloroquine (HCQ) analysis was to evaluate the effectiveness of HCQ with or without Azithromycin (Azith) in treating SARS-CoV-2 infection. To answer this question, we designed our study cohort based on criteria that might be utilized in a clinical trial setting, and we weighted our risk models using propensity for receiving treatment.

As of April 30, 2020 there were 7,193 COVID-19 positive cases identified in the VA out of roughly 64,000 individuals tested overall.^1^ The case definition dates and data available as of April 30^th^ were used to define the base cohort for these analyses, as use of HCQ virtually ceased at this time following the pharmacy benefits management (PBM) and Federal Drug Administration (FDA) revocation of HCQ emergency use authorization.

Index date for all outcomes was 48 hours after the date of VA hospital admission that occurred on the same day or up to 21 days after the first positive COVID-19 test date. To avoid immortal time bias, all subjects in the cohort required a positive SARS-CoV-2 test, and hospitalization without mechanical ventilation, discharge or death within 48 hours of admission. Following additional exclusion of individuals with prior exposure to HCQ or Azithromycin, the final analytic sample consisted of 1,733 patients.

All-cause mortality information was available through the VA mortality data based on BIRLS, clinical records and social security death index data. We considered 30-day mortality, not restricted to in-patient deaths. Individuals who died more than 30 days after hospital admission were censored at 30 days, along with those who never had the event of interest. Time-to-death was quantified in days from the index date, with the relationship between treatment and death being interpreted as the average treatment effect had each individual remained on their prescribed treatment.

For intubation or the ‘mechanical ventilation’ outcome, we considered only invasive ventilation as identified through ICD-10 and CPT procedure codes. We analyzed the outcome using time-to-intubation within 21 days of hospital admission, with censoring at death or discharge. We allowed 21 days to intubation to reduce potential bias related to residual confounding and censored at discharge to avoid capturing any intubations from subsequent/unrelated hospitalizations following discharge.

Propensity scores for each treatment were converted to a stabilized inverse propensity of treatment weight (sIPTW) by multiplying the reciprocal of propensity score for treatment received by the frequency of the treatment. These sIPTWs were used as subject-level weights in our Cox proportional hazards models for estimating treatment effects on 30-day mortality and 21-day intubation. We used multiple imputation by chained equations for all missing variables and combined the 10 imputed sets using Rubin’s rules at the end of all analyses.^2^

Of the 429 individuals treated with both HCQ and Azith in the first 48 hours following VA hospital admission, who remained alive in the hospital after 48 hours, 90 (21%) died within 30 days after admission and 64 (15%) were intubated within 21 days of admission. After weighting, those on both treatments had a 18% increased hazard of dying within 30 days (HR=1.18, 95% CI: 0.88, 1.58) and 54% increased hazard of being intubated within 21 days (HR=1.54, 95% CI: 1.07, 2.23), compared to those on neither treatment within the first 48 hours after hospitalization (N_treated_=737, N_died30_=141 (19%), N_intub21_=69 (9%)).

The results from our, independent, analyses indicate that there were increased risks for 30-day mortality and 21-day intubation outcomes amongst COVID-19 positive Veterans treated with HCQ, with or without Azith, in the first 48 hours following hospitalization. While most of these results were not statistically significant, the direction of the effect was consistent across all models, and comparable to other studies of HCQ for the treatment of SARS-CoV-2 infection in the inpatient hospital setting.

**VA Figure 1. Study design, assessment windows, and confounders**


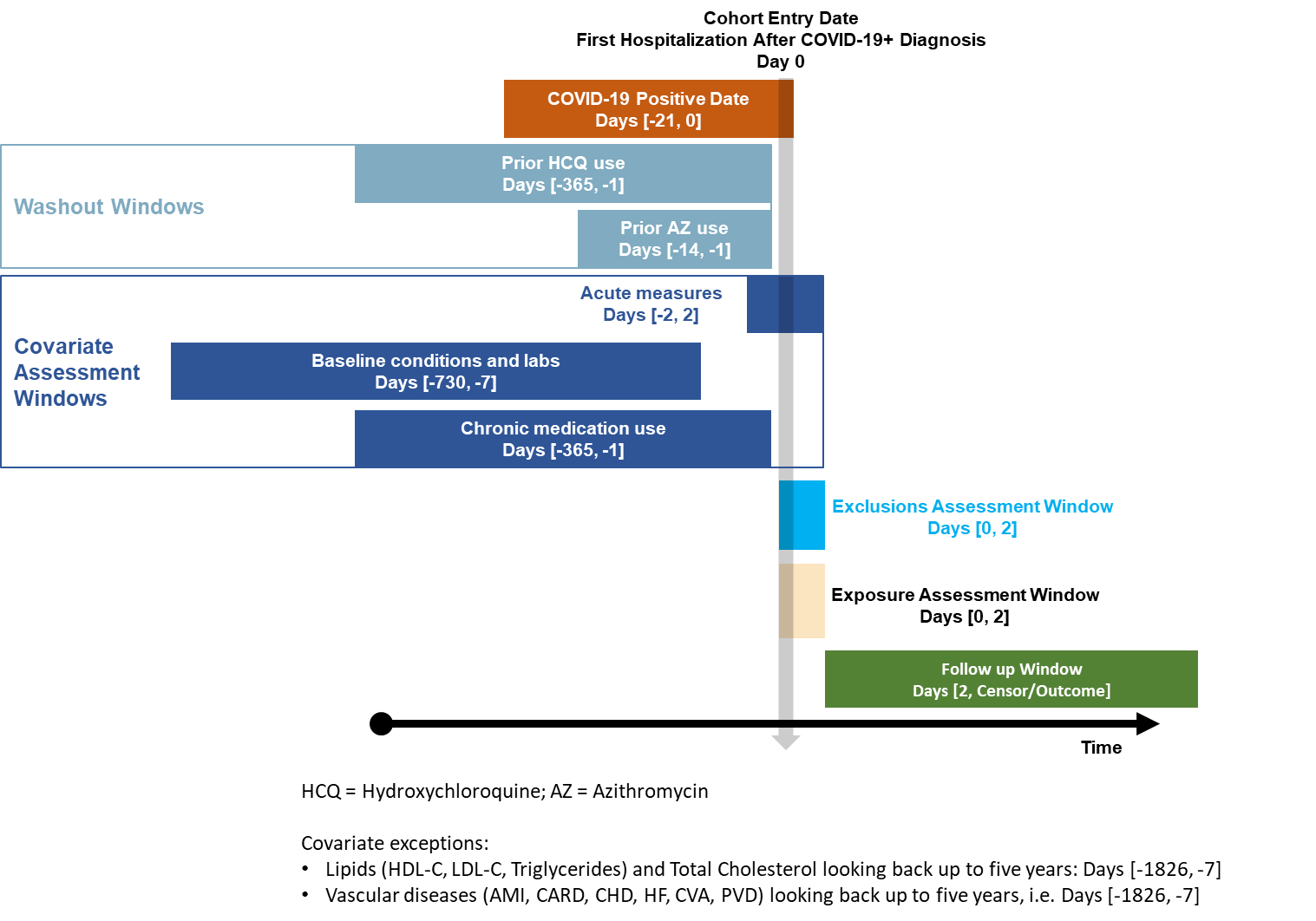


1. COVID-19 National Summary - VA Access to Care. U.S. Department of Veterans Affairs. https://www.accesstocare.va.gov/Healthcare/COVID19NationalSummary. Accessed August 8, 2020.
2. White IR, Royston P, Wood AM. Multiple imputation using chained equations: Issues and guidance for practice. Stat Med. 2011 Feb 20;30(4):377-99. doi: 10.1002/sim.4067. Epub 2010 Nov 30. PMID: 21225900. <https://pubmed.ncbi.nlm.nih.gov/21225900/>
